# Visual Outcomes and Prognostic Factors of Traumatic Endophthalmitis Treated by Pars Plana Vitrectomy: 11 Years Retrospective Analysis

**DOI:** 10.3390/jcm12020502

**Published:** 2023-01-07

**Authors:** Mădălina-Claudia Hapca, Ștefan Cristian Vesa, Simona-Delia Nicoară

**Affiliations:** 1 Doctoral School of Medicine, “Iuliu Hațieganu” University of Medicine and Pharmacy, 8, V. Babeș Str., 400012 Cluj-Napoca, Romania; 2 Department of Pharmacology, Toxicology and Clinical Pharmacology, “Iuliu Hațieganu” University of Medicine and Pharmacy, 400012 Cluj-Napoca, Romania; 3 Department of Ophthalmology, “Iuliu Hațieganu” University of Medicine and Pharmacy, 8, V. Babeș Str., 400012 Cluj-Napoca, Romania; 4 Ophthalmology Clinic, Emergency County Hospital, 3–5 Clinicilor Str., 400006 Cluj-Napoca, Romania

**Keywords:** eye injuries, pars plana vitrectomy, traumatic endophthalmitis, risk factors

## Abstract

Aim: To evaluate the visual outcome of traumatic endophthalmitis and describe the risk factors associated with poor visual acuity and retinal detachment (RD) development over an 11-year period. Methods: Medical records of 34 patients with traumatic endophthalmitis who underwent PPV over a period of 11 years (1 January 2010–31 December 2020) were reviewed. We extracted details regarding demographic data, initial and final best corrected visual acuity (BCVA) using a standard Snellen chart, wound and IOFB characteristics, ocular associated lesions, and treatment. The outcome was evaluated according to the final BCVA which was defined as poor < 0.1 or good ≥ 0.1 Results: Endophthalmitis rate was 29.8% in open globe injuries. The mean age was 43.6 ± 16.5 years and the majority of patients were males (32 out of 34, 94.1%). All patients received systemic (moxifloxacin) and intravitreal antibiotherapy. We performed pars plana vitrectomy (PPV) in all cases. Poor visual outcome was associated with wound size ≥ 3 mm (*p* = 0.02), the association of IOFB (*p* = 0.016), and the development of RD (*p* = 0.00). The presence of IOFB (*p* = 0.01) and wound size ≥ 3 mm (*p* = 0.01) were statistically associated with RD development. After treatment, 47.05% of patients achieved final BCVA ≥ 0.1. Conclusion: Wound size ≥ 3 mm, IOFB and RD were risk factors for poor visual outcomes in traumatic endophthalmitis.

## 1. Introduction

Endophthalmitis is a severe inflammation of the intraocular tissue due to infection that can lead to significant visual loss [1]. The infection occurs as a result of the inoculation of microorganisms in the eye, either following surgery or trauma (classified as exogenous), or from the hematogenous bacterial spread (classified as endogenous) [2]. Post-traumatic endophthalmitis is a severe complication of penetrating eye injuries [3]. It occurs in 4–16% of open-globe injuries and represents 25–30% of all cases of endophthalmitis [4]. Post-traumatic endophthalmitis is typically associated with worse visual outcomes compared to other types of endophthalmitis due to a variety of factors, such as the microorganisms involved, which are distinct and more virulent, higher likelihood of delayed diagnosis and initiation of treatment, coexisting traumatic eye injuries, the possible association of an intraocular foreign body (IOFB) or lens rupture and large wound size [3,5,6]. Intravitreal antibiotics and pars plana vitrectomy (PPV) represent the mainstay treatment of post-traumatic endophthalmitis [7]. Although many factors are responsible for visual prognosis, advances made in vitreoretinal surgery significantly improved the final anatomical and functional outcome. In this study, we evaluated all post-traumatic endophthalmities who underwent PPV over an 11-year period with the aim to identify the relationship between the clinical features and visual outcome and outline the risk factors associated with poor prognosis.

## 2. Materials and Methods

### 2.1. Study Design and Subjects

During an 11 year period, from 1 January 2010 to 31 December 2020, the charts of all patients diagnosed with post traumatic endophthalmitis and treated by PPV in the Department of Ophthalmology, Emergency County Hospital, “Iuliu Hațieganu” University of Medicine and Pharmacy from Cluj-Napoca, Romania were reviewed and included in this study. The study was conducted in accordance with the Declaration of Helsinki and was approved by The Ethics Committee belonging to the “Iuliu Hațieganu” University of Medicine and Pharmacy (number 163/20 June 2022) and by the Institutional Review Board of Cluj County Emergency Clinical Hospital (number 79/18 April 2022) and all patients signed the informed consent. In all cases, the following data were extracted from medical records: age, gender, rural or urban setting of the eye trauma, initial and final BCVA (Snellen Chart), ocular injury features (type of accident, injury location, lens breach, hyphema, tissue prolapse), the time elapsed between injury and diagnosis of endophthalmitis, surgical gestures (timing of primary repair and PPV, number of surgeries and intravitreal antibiotic injections) presence or absence of IOFB and complications. In cases with IOFB, information regarding the location, material and size was collected. Due to economical reasons, we did not perform routine cultures for all cases. Visual outcome was defined according to final BCVA as poor < 0.1 or good ≥ 0.1.

In our study, the diagnosis of post-traumatic endophthalmitis was established based on detailed history regarding previous ocular injury, symptoms (impaired or blurred vision, intense eye pain that worsened with time, photophobia, floaters and tearing) and slit lamp examination (intense conjunctival hyperemia, chemosis, anterior chamber reaction with hypopion, corneal edema and opacities, vitreous opacities). We included in the study only patients with a clinical diagnosis of post traumatic endophthalmitis. The diagnosis was confirmed either at initial presentation or during follow-up in patients who had previously undergone primary repair of penetrating ocular injury or PPV for IOFB extraction. A complete examination of both eyes was carried out and in all cases with suspicion, orbital radiography and/or computer tomography were performed to certify or rule out an IOFB. During this 11-year period, 34 cases fell into the above-mentioned criteria.

### 2.2. Treatment

All patients received immediate systemic antibiotics (moxifloxacin 400 mg daily for 5 days) and a topical combination of antibiotic and steroid therapy (tobramycin + dexamethasone). In all cases with open wounds, the primary surgical repair of leaking wounds was carried out as soon as possible, within 24 h from admission, except in cases with delayed presentation. When present, the repositioning or excision of prolapsed uveal tissue was performed at the same time as primary repair.

The intravitreal antibiotic regimen included either vancomycin 1 mg/0.1 mL alone, or vancomycin 1 mg/0.1 mL combined with ceftazidime 2.2 mg/0.1 mL or amikacin 0.4 mg/0.1 mL, according to the severity of the disease. In all cases, intravitreal dexamethasone 0.4 mg/0.1 mL was added to the regimen. All IOFB extractions were performed primarily in conjunction with open wound repair. If an IOBF was present, a core vitrectomy was carried out and after releasing the foreign body by lysis of all its adhesions with the vitreous and widening the sclerotomy according to IOFB size, it was extracted with the intraocular magnet. In cases where signs of endophthalmitis developed after the primary repair or IOFB removal, either a second PPV with intravitreal antibiotics and dexamethasone or intravitreal injections alone was performed. All cases were operated on by the same experienced vitreoretinal surgeon (SDN).

Additional surgical procedures were performed according to the status of the patient’s eye as follows: primary lensectomy/phacoemulsification if cataract or lens dislocation was present; endolaser photocoagulation if retinal lesions were identified; fluid/air exchange, followed by laser or cryotherapy to seal the retinal break and silicone oil tamponade, if retinal detachment (RD) was associated.

### 2.3. Statistical Analysis

Data were analyzed using the MedCalc^®^ Statistical Software version 20.104 (MedCalc Software Ltd., Ostend, Belgium; https://www.medcalc.org, accessed on 20 October 2022). All numerical data are expressed either as the median (25–75 percentiles) or as the mean ± standard deviation, depending on the normality of distribution (tested with the Shapiro–Wilk test). Categorical data were expressed as frequency and percentage. The outcome was evaluated according to the final BCVA: poor < 0.1 or good ≥ 0.1. In comparative statistical analysis, categorical data were assessed by the Chi-square test or Fisher test. Univariate logistics regression was applied to examine the associations between risk factors and the final BCVA. A *p*-value of less than 0.05 was considered statistically significant throughout the study.

## 3. Results

During a period of 11 years, 34 consecutive patients were treated by PPV for traumatic endophthalmitis by the same surgeon (SDN) in our department. The total number of open globe injuries that underwent PPV during the same period of time and managed by the same surgeon was 114 (57 cases with IOFB and 57 cases without IOFB), giving a rate of traumatic endophthalmitis of 29.5%. The majority of patients were male (94.1%) with a mean age of 43.6 years (±16.5). Among these, 16 patients (47.1%) had trauma to the right eye and 17 (52.9%) to the left eye; no patient reported wearing eye protection at the time of injury. The demographic characteristics of the patients are shown in Table 1. We found no significant correlation between the demographic data and the final visual outcome.

In our study, post-traumatic endophthalmitis was diagnosed based on symptoms and characteristic clinical findings following open-globe injuries (OGI). The ocular symptoms were found in all our patients and were represented by worsening eye pain and decreasing BCVA. Table 2 summarizes the common clinical aspects typical of post-traumatic endophthalmitis among the subjects in our study.

The clinical signs were present at the initial examination in 27 cases (79.4%), whereas in the remaining seven cases (21.6%), they developed after the IOFB extraction or primary wound closure, either within the hospitalization period (5 cases), or at follow up (2 cases). Among the seven cases who developed endophthalmitis at a later stage, three patients underwent primary PPV for IOFB removal with no intraoperative signs of infection. They were treated with intravitreal antibiotics with no additional PPV. In all other 31 cases, vitreous exudation was noted during PPV and in addition, five patients displayed retinal exudates and infiltrates.

All patients in our case series reported a decrease in BCVA after trauma. At the initial consultation, six patients (17.64%) had BCVA ≥ 0.1 and 25 (73.5%) had BCVA less than counting fingers (CF). After surgery, 23 (67.64%) patients achieved final BCVA of CF or more with 16 cases (47.05%) ≥ 0.1, six cases (17.64%) < 0.1 and one case (2.94%) CF. No patient ended up with a final BCVA of NLP and no evisceration/enucleation was performed in our series. A summary of the visual outcome is illustrated in Table 3.

The ocular features of the injuries are presented in Table 4. The maximum time interval between the moment of trauma and the development of endophthalmitis was 35 days in our series (mean ± SD; 2.23 ± 6.79). Of the 34 patients, 21 (61.67%) had self-sealing wounds and a history of penetrating ocular trauma and 13 cases (38.23%), had open globe injuries of which five had underwent primary closure at the referring facility. In order to quantify the time interval between the moment of trauma and the primary repair and to be able to perform the statistical analysis, we included all the self-sealing wounds within the first 24 h category. The interval of time from injury to hospital presentation was 2.4 ± 2.1 days (range, 0–8 days) excluding four patients due to incomplete information. The mean ± SD duration from trauma to PPV was 10.14 ± 20.49 (range, 0–120 days). The mean ± SD duration of admission was 11.76 ± 6.57 days (range, 3–29 days).

Corneal and scleral lesions were identified in 19 (55.88%) and 15 (44.11%) patients respectively. RD, vitreous hemorrhage, hyphema, tissue prolapse, and traumatic cataracts were detected in 12 (35.3%), 8 (23.5%), 6 (17.6%), 3 (8.8%) and 15 (44.1%) cases, respectively. Out of 15 cases (44.1%) of traumatic cataract, 10 cases (66.6%) had diffuse cataract, 2 (13.3%) cases had lens material in the vitreous cavity and 3 cases (20%) had localized lens capsule disruption with overlying cataract.

IOFB was present in 17 cases (50%) of post-traumatic endophthalmities and it was removed by PPV in all of them. All 17 IOFBs were metallic, of which 16 were magnetic and 1 non-magnetic. The average IOFB length was 3.73 ± 1.95 mm (range, 1–7 mm). Among these cases, a total of 13 eyes (76.5%) had a poor visual outcome and only 4 obtained a final BCVA ≥ 0.1. IOFB was retinal in 10 cases (58.8%) and vitreal in 7 cases (41.2%). Of the 10 patients with retinal IOFBs, only 1 gained final BCVA ≥ 0.1. Endophthalmitis was clinically evident at the time of presentation in 13 IOFB cases, whereas in the remaining four cases, it occurred 1 day, 2 days, 4 days, and 35 days after the IOFB removal respectively. The presence of an IOFB was statistically associated with worse outcomes (*p* = 0.016).

RD occurred in 12 cases (35.3%). Among these cases, 10 had IOFB with the retinal location. Endophthalmitis was diagnosed at presentation nine 9 cases and it developed after IOFB removal in two cases and after primary wound closure in one case. The zone of injury was corneal in seven cases and scleral in five cases. The status of the retina could be evaluated at the initial examination in 6 of the 34 cases (17.6%) and none of them presented RD at that time. We identified RD during initial surgery in five cases, three of them with IOFBs. RD was detected postoperatively in seven cases with previous IOFB extraction. Risk factors for RD in our series were wounds larger than ≥3 mm (*p* = 0.012) and the presence of IOFB (*p* = 0.012). Wound location (corneal or scleral) did not influence the risk of RD (*p* = 1.000).

Visual outcome was compromised in all cases of RD. Extensive retinal lesions and retinal necrosis were seen in seven cases, postoperative recurrent RD that required a second PPV was noted in two cases and RD repair with silicon oil tamponade was performed in three eyes. In total, nine patients had a final BCVA of HM or less, one patient had CF and two patients had 0.06.

The most common cause of penetrating globe injury was metal in 29 cases (85.3%). Other causes were wood in three (8.8%), thorns in two (5.9%), and one traumatic wound dehiscence in a patient with a corneal transplant who fell down the stairs (2.9%).

Table 5 illustrates the relationship between various factors and the final BCVA. The results indicated that wounds larger than 3 mm (*p* = 0.002), IOFB (*p* = 0.017) and RD (*p* = 0.000) had significantly worse outcomes. No statistically significant differences were observed for the rest of the analyzed parameters.

## 4. Discussion

Post-traumatic endophthalmitis is a serious complication of penetrating ocular trauma. It represents one of the main causes of unilateral vision loss among young adults, which is why it requires urgent diagnosis and treatment [8].

It is known that post-traumatic endophthalmitis is often associated with an unfavorable visual prognosis which is linked to a variety of socioeconomic problems and the impairment of psychological well-being [8,9,10]. When compared to healthy subjects, patients with traumatic endophthalmitis and the poor visual outcome had a low quality of life [9,11], economic decline [11], and increased psychological symptoms [9]. Other aspects of the impact that visual impairment has on these patients include: the medical situation implying expensive services and hospitalizations, frequent follow-ups, and loss of patients’ income; the functional situation that may require vocational rehabilitation and special education and the socioeconomic aspect indicating the high cost of work capacity’s loss [8]. Patients at risk for traumatic endophthalmitis are active relatively young men because they employ more frequently in high-risk activities [10,12].

The incidence of post-traumatic endophthalmitis varies widely, between 3.1–48.1% [3,4,13,14]. It was reported that the incidence may also differ depending on the mechanism of trauma: 3.1–11.9% in open globe injuries in the absence of IOFB and rising at 3.8–48.1% in the presence of IOFB [3,15]. During 11 years, we managed 114 cases of OGI that required PPV, of which 57 (50%) had IOFB. The frequency of endophthalmitis within the two categories was equal in our study, 29.8% (17 cases of 57 IOFB and 17 cases of 57 penetrating injuries). The frequency among penetrating injuries without IOFB is higher in our series as compared to the literature [3,5,14]. We explain this higher figure with two facts: firstly, we included in the study only patients who were operated on by the same surgeon, which does not reflect the total number of OGIs, and secondly, we are a tertiary care center. The rate of endophthalmitis within the subgroup of patients with IOFBs matches the one cited in the literature [3,5,16].

Retained IOFBs increase the risk of endophthalmitis [3]. In our case series, IOFBs were present in 17 patients (50%) and all of them were metallic. The nature of the IOFB may influence the incidence of endophthalmitis [3,5,17,18,19]. Jonas et al. reported that organic IOFBs had a higher tendency to develop endophthalmitis compared to metallic ones [17]. On the contrary, military members who sustained OGI with IOFB during the war did not show an increased risk of endophthalmitis even if removed late, because in the military setting, IOFBs may self-sterilize due to their high speed and temperature [20]. Significant differences in regard to visual outcome are found between anterior and posterior segment IOFBs, with the latter having a worse prognosis because they are more likely to produce RD [21,22,23]. All of the 17 cases were located posteriorly and 10 of them (58.8%) developed RD. We found that the presence of an IOFB is an important factor contributing to poor visual outcomes (*p* = 0.016). Final BCVA ≥ 0.1 was achieved only in 4 patients (23.5%), with the remaining 13 ones ending up with CF < 0.1 in two cases, CF in one case, HM in six cases, and LP in three cases. Notably, of 17 eyes without IOFBs, 12 achieved BCVA ≥ 0.1 (70.6%). The timing of IOFB removal is controversial. Some authors found that delaying surgery by more than 24 h increases the risk of developing endophthalmitis and proliferative vitreo-retinopathy [17,19,21,24,25]. In contrast, other studies in which there was a delay of 1 day to 3 years in surgery did not find any additional risk of endophthalmitis [22,26]. We performed PPV in the first 24 h in three cases of endophthalmitis with IOFB (range, 0–8 days). In all cases of IOFB complicated with endophthalmitis, surgery must be performed as soon as possible.

The incidence of positive cultures in different studies varies between 17–81% [13,27,28]. Microbiological results indicated that the most common agents responsible for the infection are bacteria, representing approximately 80–90% of all positive cultures [29,30], and on occasion, fungi [2,4]. The most common isolates among bacteria and fungi are Gram-positive species such as *Staphylococcus*, *Streptococcus* and *Bacillus* and *Candida*, *Aspergillus* and *Fusarium*, respectively [5,29,30]. Of these, *Bacillus endophthalmitis* is noteworthy, as it can lead to the total destruction of the eye tissues within 24 h–48 h despite treatment [31]. In our study, cultures were not routinely performed and the diagnosis was made based on past ocular trauma history and clinical evaluation. There were a total of six culture samples, of which three were culture-negative, two were positive for *Staphylococcus aureus* and onefor *Enterococcus*. As mentioned previously, culture negativity in the presence of clinical signs of endophthalmitis does not rule out the diagnosis, nor is it necessary [4]. On the contrary, culture positivity does not always mean endophthalmitis. In one study, 33% of patients with penetrating ocular trauma had positive cultures and none of them developed endophthalmitis [32]; 26% of eyes with IOFB were culture positive in a series report and no sign of intraocular infection was noted [33]. In our opinion, the diagnosis of endophthalmitis is mainly clinical and culture results should always be corroborated with ocular signs and symptoms.

The initial step in managing an OGI is primary wound closure. It is recommended to be performed as soon as possible, within the first 24 h after trauma [34]. Delaying primary wound repair more than 24 h increases the risk of developing endophthalmitis regardless of the absence [35] or the presence of an IOFB [3,17,24]. The risk of endophthalmitis also increases significantly if systemic antibiotic therapy is initiated after the first 24 h [36]. Zhang et al. noted that the risk of infection increases with each hour’s delay and there is no safety in delaying the surgery [35]. Due to the fact that we are a tertiary care unit, primary repair within 24 h in our case series was possible in only four cases; we had one case with a self-sealing wound in which PPV was carried out within 24 h and achieved final BCVA ≥ 0.1. Because of the extent of damage caused by the injury itself, none of the four patients achieved a good visual outcome; two of the four patients exhibited IOFB with PPV conducted on the same day and both of them developed RD; the remaining two cases had corneal wounds ≥ 7 mm and endophthalmitis was diagnosed following primary suture on day 5 and day 15, respectively, at which time intraocular antibiotics were administered followed by PPV 5 days afterward in both cases. In our series, there was no statistical correlation between the early (<24 h) wound closure and final BCVA. The low rate of wound closure within 24 h from trauma may be explained by the late referral from other hospitals and failure to see an ophthalmologist, most of our patients, 25 (73.5%) come from rural areas. Other studies also found that patients injured in rural areas were at increased risk for post-traumatic endophthalmitis, not only because of difficult accessibility, but also due to the higher aggressivity of germs in the rural setting [5].

In terms of the wound site, the corneal lesions accounted for 55.9% (19 eyes) of total and scleral ones for 44.1% (15 eyes), thus the corneal site was more frequently associated with endophthalmitis which is similar to other studies [14,37]. Penetrating corneal injuries are thought to be a higher risk for endophthalmitis because of the more effective inoculation of microorganisms [37]. The length of the scleral wound was ≥3 mm in all seven cases with IOFB and in only three of the eight cases without IOFB. In contrast, in corneal lesions, the wound length was ≥3 mm only in three of the 10 cases with IOFB and in three of nine cases without IOFB. Our results do not match the ones found by Faghihi et al., who reported a higher incidence of endophthalmitis in cases with IOFB and small wounds (2–3 mm) [37]. Schmidseder also found that smaller wounds exhibited a higher endophthalmitis risk [36]. Cases with wound length ≥ 3 mm had poorer visual outcomes compared with wounds ˂ 3 mm (*p* = 0.002) in our series. Of the 17 patients within this category, 14 (82.4%) ended up with BCVA < 0.1. One major factor that contributed to this prognosis was RD which was present in 10 (58.8%) of the 17 eyes. None of the threepatients with wound size ≥ 3 mm and final BCVA ≥ 0.1 developed RD.

According to the literature, lens breach during ocular trauma may promote organisms’ multiplication by several mechanisms: direct access inside the eye, decreasing aqueous flow and clearance of organisms, and nutritional support [3,38]. The results of several studies are inconsistent; some authors observed an increased risk of endophthalmitis associated with lens breach [5,38,39] and others found it to be insignificant [25,35,40]. Lens breach did not have an impact on the outcome in our series (*p* > 0.05).

The effect of intraocular tissue prolapse and hyphema in endophthalmitis development is still disputed. Some authors claim that tissue prolapse increases the risk of endophthalmitis due to prolonged exposure to organisms [40]. Gupta et al. found no effect of tissue prolapse [41]. Contrarily, in other reports, tissue prolapse and hyphema were independent protective factors against post-traumatic endophthalmitis [35,37]. One mechanism explaining the protective role of hyphema against infection is by the disruption of the blood ocular barrier and release of inhibitory bacterial growth factors in the anterior chamber; uveal tissue, instead, directly blocks the entry of bacteria into the eye [37]. Tissue prolapse and hyphema did not influence the visual outcome in our series (*p* > 0.05).

A severe complication that can develop in up to 25% of traumatic endophthalmities is RD [7,10,42]. Visual outcome in cases with RD after or during the course of traumatic endophthalmitis is significantly worse compared with primary RD, with up to two-thirds of cases reaching a BCVA of only 20/400 after surgical repair [43]. The incidence of RD in our series was 35.3% (12 cases), higher than cited in other studies [7,42]. Visual outcome was significantly worse (*p* = 0.00), with none of the cases reaching final BCVA ≥ 0.1. Visual acuities in RD cases were LP in three patients, HM in six cases, CF in one case, and 0.06 in two cases. The presence of IOFB (*p* = 0.01) and wound size ≥ 3 mm (*p* = 0.01) were statistically associated with RD development in our study. PPV in the presence of endophthalmitis is more challenging and therefore associated with a higher risk for RD [8]. Mechanical manipulation of the vitreous during surgery and vitreous tap can produce retinal breaks, especially if retinal necrosis is present [44]. The visual prognosis is better in cases of delayed onset RD than in those concomitant with the infection [3]. Endophthalmitis is also more difficult to treat in the presence of RD [3]. If RD repair is made during the acute stage of endophthalmitis, the priority is to treat the infection [45]. After vitrectomy, fluid air exchange, and retinal break seal, a decision regarding internal tamponade and intravitreal antibiotic therapy must be made. Silicon oil is preferred in these situations due to its proven antimicrobial activity [46]; it may also help in reducing recurrent RD and can be left in place indefinitely until the infection subsides [45,47]. Some authors state that in cases with silicon oil or gas, a half-dose of intravitreal antibiotic should be administered because it will diffuse only in the space unoccupied by oil or gas, and the risk of retinal toxicity increases [3]. However, in the treatment of endophthalmitis with concurrent RD, the benefit of properly treating and eliminate the infection outweighs the risk of toxicity [45,48]. In cases of delayed onset of RD, PPV is generally indicated with intravitreal antibiotic re-administration if infection persists [45].

Unlike the postoperative endophthalmitis in which the Early Vitrectomy Study specifies therapeutic guidelines, there is no standard therapeutic protocol for post-traumatic endophthalmitis [4]. Primary wound closure and IOFB removal are recommended within the first 24 h in all OGIs. Intravitreal antibiotic treatment should be the initial step in the management of all traumatic endophthalmities [3]. Some authors consider that all cases should undergo early therapeutic PPV [35,49]. The rationale behind this strategy relies on a principle from general surgery, “Ubi pus ibi evacua”, meaning that where there is pus, it must be evacuated. However, timing is an essential consideration in the treatment algorithm. Some studies suggest that not all traumatic endophthalmitis require PPV and that intravitreal therapy alone may be effective in cases with mild infection and early presentation (<24 h) [3,14]. If no improvement or clinical deterioration is noted within 48 h despite intravitreal antibiotics, PPV should be considered [3]. In addition, PPV should be performed as soon as possible in high-risk cases such as soil contamination, IOFB, delayed presentation (>24–48 h), or retinal detachment.

The route by which effective concentrations of intraocular antibiotics are reached is through intravitreal injection [50]. Because treatment should commence as early as possible, most often it is empirical, before the results of the lab tests are available [51]. Gram-positive and Gram-negative bacteria are the most common, therefore treatment should cover both. Vancomycin (1 mg/0.1 mL) for Gram-positive combined with ceftazidime (2.25 mg/0.1 mL) for Gram-negative are the most recommended first-line intravitreal antibiotics [3]. Amikacin (0.4 mg/0.1 mL) is an alternative to ceftazidime in penicillin-allergic patients, but caution is required due to retinal toxicity [3,5]. In addition to intravitreal therapy, in 58.8% of cases, we added systemic corticosteroids, depending on the degree of ocular inflammation. Conrady et al. found no significant outcome regarding final BCVA in patients who received systemic steroids vs. those who did not; still, they found that systemic steroids lower the risk of enucleation or evisceration in severe cases [52]. Likewise, no evisceration or enucleation was needed in our case series. Most systemically administered antibiotics do not reach satisfactory intraocular concentrations to successfully treat infection [50]. However, fourth-generation fluoroquinolones, moxifloxacin, and gatifloxacin, can reach the vitreous cavity when administered systemically and achieve effective concentrations against the majority of bacteria involved in traumatic endophthalmitis [3]; besides, the effectiveness of systemic antibiotherapy may be enhanced by the malfunctioning of the ocular barriers in the inflammatory setting. In our series, 94.1% (32 patients) received oral moxifloxacin and 58.8% (20 patients) were given systemic corticosteroids after having undergone antibiotic treatment for 24 h.

PPV in the setting of traumatic endophthalmitis can be very challenging due to poor visualization caused by underlying eye lesions and inflammation. There are several significant benefits of PPV in endophthalmitis: it removes toxin-producing bacteria and inflammatory cells, clears the media by eliminating the vitreous membranes causing traction and RD, allows specimen culture, and provides better perfusion of intravitreal antibiotics. Vitreous is a culture medium that promotes the growth of bacteria. Following the injection of intravitreal antibiotics, bacteria lyse and release toxins, therefore the PPV performed after 24–48 h is clearing these cells [3]. However, in our series all IOFB cases underwent PPV within 24 h from admission; 11 of them received intravitreal antibiotics at the end of surgery, and the remaining six at a later stage. In penetrating injuries without IOFB (17 eyes), PPV with intravitreal antibiotics at the end of surgery was performed in nine cases and intraocular antibiotics were first followed by PPV in eightcases. Notably, eight (88.9%) of the nine cases with combined PPV and antibiotics achieved BCVA ≥ 0.1, whereas only four (50%) of the eight cases with intraocular antibiotics first and PPV at a later stage, ended up with BCVA ≥ 0.1. Although it did not reach statistical significance (*p* = 0.07), combined PPV with intraocular antibiotics at the end of surgery provided a better prognosis for the patients in our series. Because the number of cases is small and the visual outcome is influenced by a variety of factors, in order to draw a firm conclusion on this aspect more randomized clinical trials should be conducted.

To date, there are no controlled clinical trials to prove the effectiveness of intravitreal or systemic prophylaxis in traumatic endophthalmitis. Under normal conditions, the blood-ocular barrier does not allow systemic antibiotics to enter the vitreous cavity. Nevertheless, in the setting of traumatic endophthalmitis, the function of this barrier is impaired, allowing antibiotics to penetrate better inside the eye [31]. In recent years, fluoroquinolones have been the most commonly used antibiotics for prophylaxis [3,4,14]. Moxifloxacin in particular has been shown to be effective against most Gram-positive and Gram-negative bacteria, including *Bacillus* species [15]. Some studies failed to observe any decrease in the incidence of infection despite systemic prophylaxis [5,38]. A more recent study showed a 3% endophthalmitis rate after OGI with either intravenous cefazolin and oral ciprofloxacin, or oral cefuroxime and oral ciprofloxacin [53]. Intravitreal antibiotic prophylaxis has been proven to be useful in animal models [54], but its utility in clinical practice remains unclear. However, there are several studies that showed a lower incidence of posttraumatic endophthalmitis with intravitreal antibiotic prophylaxis [33,40,55]. Mieler et al. had no case of endophthalmitis in their study population after having selectively administered intraocular antibiotics in high-risk cases [33]. Abouammoh et al. compared two cohorts, one that included prophylactic intravitreal antibiotics and one that did not, and found that endophthalmitis rates were higher in the absence of prophylaxis [55]. On the other hand, Colyer et al. and Ehlers et al. reported a low incidence of endophthalmitis in OGI without any intravitreal prophylaxis [20,56]. In all cases of OGI, without clinical signs of endophthalmitis, we administer systemic antibiotics (moxifloxacin) either orally or intravenously, but not intravitreally.

In our series, three factors were associated with poor visual outcome: wound size ≥ 3 mm, IOFB, and development of RD. We also evaluated other factors that could possibly influence the outcome such as age, initial VA, wound location, tissue prolapse, vitreous hemorrhage, lens breach, and primary closure but none of them were significantly correlated with final BCVA.

The limitations of this study are the small sample size, absence of cultures in all cases, its retrospective design, lack of a defined protocol treatment, and the variability of presentations. Besides, because we are a tertiary care center, the severity of our study population may be higher.

## 5. Conclusions

The posttraumatic endophthalmitis rate on our OGIs over a period of 11 years was 29.8%. Wound size ≥ 3 mm (*p* = 0.02), the association of IOFB (*p* = 0.016), and the development of RD (*p* = 0.00) significantly worsened the visual outcome of the patients in this series. The risk factors for RD development were the presence of IOFB (*p* = 0.01) and wound size ≥ 3 mm (*p* = 0.01).

## Figures and Tables

**Table 1:** Demographic characteristics.

Variable		BCVA < 0.1	BCVA ≥ 0.1	*p* Value
Age	<50 (*n* = 22) ≥50 (*n* = 12)	13 (72.2%) 5 (27.8%)	9 (56.2%) 7 (43.8%)	0.540
Gender	Female (*n* = 2) Male (*n* = 32)	2 (11.1%) 16 (88.9%)	- 16 (100%)	0.487
Eye	Right (*n* = 16) Left (*n* = 17)	9 (50.0%) 9 (50.0%)	7 (43.8%) 8 (56.2%)	0.984
Eye protection	Yes (*n* = 0) No (*n* = 34)	- 18 (100%)	- 15 (100%)	
Location	Rural (*n* = 25) Urban (*n* = 9)	15 (83.3%) 3 (16.7%)	10 (62.5%) 6 (37.5%)	0.250

BCVA: Best corrected visual acuity.

**Table 2:** Clinical findings among endophthalmitis cases.

Clinical Finding	Present *n* (%)	Absent *n* (%)
Hypopion	22 (64.7)	12 (35.3)
Corneal edema	22 (64.7)	12 (35.3)
Keratic deposits	6 (17.6)	28 (82.4)
Conjunctival hyperemia	34 (100)	0 (0)
Tyndall effect	26 (76.5)	8 (23.5)
Pupillary fibrin membrane	15 (44.1)	19 (55.9)

**Table 3:** Visual outcome in patients with post-traumatic endophthalmitis.

VA	Preoperative BCVA *n* (%)	Postoperative BCVA *n* (%)
≥0.1	6 (17.64)	16 (47.05)
<0.1	3 (8.82)	6 (17.64)
CF	1 (2.94)	1 (2.94)
HM	17 (50)	7 (20.58)
LP	6 (17.64)	4 (11.76)
NLP	1 (2.94)	0
Total	34	34

VA: Visual acuity; BCVA: Best corrected visual acuity; CF: Counting fingers; HM: Hand motion; LP: Light perception; NLP: No light perception.

**Table 4:** Clinical features of patients with traumatic endophthalmitis.

Clinical Feature		No. of Eyes (%)
Wound location	Corneal Scleral	19 (55.9) 15 (44.1)
Wound Size	<3 mm ≥3 mm	17 (50.0) 17 (50.0)
IOFB	Yes No	17 (50.0) 17 (50.0)
RD	Yes No	12 (35.3) 22 (64.7)
VH	Yes No	8 (23.5) 26 (76.5)
Hyphema	Yes No	6 (17.6) 28 (82.4)
Tissue prolapse	Yes No	3 (8.8) 31 (91.2)
Lens breach	Yes No	15 (44.1) 19 (55.9)

No.: number; IOFB: Intraocular foreign body; RD: Retinal detachment; VH: Vitreous hemorrhage.

**Table 5:** Prognostic factors.

Factor	Final VA, *n* (%)		*p*-Value
	<0.1	≥0.1	
Age			0.540
<50	13 (72.2)	9 (56.2)	
≥50	5 (27.8)	7 (43.8)	
Initial VA <0.1 ≥0.1			1.000
	15 (83.3)	13 (81.2)	
	3 (16.7)	3 (18.8)	
Wound Location			1.000
Corneal	10 (55.6)	9 (56.2)	
Scleral	8 (44.4)	7 (43.8)	
Wound Size			**0.002**
<3 mm	4 (22.2)	13 (81.2)	
≥3 mm	14 (77.8)	3 (18.8)	
RD			**0.000**
Yes	12 (66.7)	0 (0)	
No	6 (33.3)	16 (100)	
IOFB			**0.016**
Yes	13 (72.2)	4 (25.0)	
No	5 (27.8)	12 (75.0)	
VH			0.233
Yes	6 (33.3)	2 (12.5)	
No	12 (66.7)	14 (87.5)	
Lens breach			1.000
Yes	8 (44.4)	7 (43.8)	
No	10 (55.6)	9 (56.2)	
Tissue prolapse			0.230
Yes	3 (16.7)	0 (0)	
No	15 (83.3)	16 (100)	
Hyphema			0.660
Yes	4 (22.2)	2 (12.5)	
No	14 (77.8)	14 (87.5)	
Timing of PPV			0.715
<48 h	6 (33.3)	4 (25.0)	
>48 h	12 (66.7)	12 (75.0)	
Primary closure			0.693
<24 h	13 (72.2)	13 (81.2)	
>24 h	5 (27.8)	3 (18.8)	
Nature of trauma			0.648
Metallic	16 (88.9)	13 (81.2)	
Non-metallic	2 (11.1)	3 (18.8)	

*n*: number; VA: Visual acuity; RD: Retinal detachment; IOFB: Intraocular foreign body; VH: Vitreous hemorrhage; PPV: Pars plana vitrectomy; Bold: *p*-value was significant.

## Data Availability

The data presented in this study are available on request to the corresponding author.
